# Magnet-Free Nonreciprocal Edge Plasmons in Optically Pumped Bilayer Graphene

**DOI:** 10.3390/nano15211622

**Published:** 2025-10-24

**Authors:** Seongjin Ahn

**Affiliations:** Department of Physics, Chungbuk National University, Cheongju 28644, Republic of Korea; sahn@cbnu.ac.kr

**Keywords:** bilayer graphene, plasmonics, Wiener–Hopf method

## Abstract

Recent theoretical studies have shown that gapped Dirac materials (such as gapped monolayer graphene) optically pumped with circularly polarized light can host edge-localized plasmon modes with nonreciprocal dispersions driven by valley population imbalance. Here, we extend this framework to Bernal-stacked bilayer graphene. Using the Wiener–Hopf method, we compute the exact edge plasmon dispersion, confinement length, and electric potential. Our results show that bilayer graphene exhibits stronger nonreciprocity in edge plasmons, requiring approximately one order of magnitude lower pump amplitude to achieve splitting compared with monolayer Dirac systems. Furthermore, the gate-tunable energy gap of bilayer graphene provides an additional degree of control, positioning optically pumped bilayer graphene as a versatile platform for magnet-free nonreciprocal plasmonics.

## 1. Introduction

Plasmons can confine light far below the diffraction limit, enabling the development of compact tunable elements for integrated photonics [1,2,3,4,5]. Breaking reciprocity so that plasmon modes preferentially propagate in one direction is particularly important for applications such as on-chip isolators and circulators [6,7,8,9,10,11]. Achieving nonreciprocal plasmon dispersion requires breaking time-reversal symmetry to lift the degeneracy between counterpropagating plasmons. The most common method of producing nonreciprocal plasmons uses static magnetic fields [12,13,14,15,16,17,18]. However, this method requires strong external magnetic fields and is incompatible with compact integrated photonic platforms [19,20,21].

Recently, alternative magnetic-free approaches to plasmonic nonreciprocity have been proposed [17,18]. In gapped Dirac materials, a nonzero Berry flux gives rise to collective edge modes with nonreciprocal plasmon dispersions in opposite propagation directions, even in the absence of an external magnetic field. This nonreciprocity occurs because the Berry curvature induces an anomalous velocity that assists or opposes charge motion along the edge. One way to induce a nonzero net Berry flux is to drive gapped Dirac materials with circularly polarized light, which selectively pumps carriers for different valleys. This valley-selective pumping leads to a population imbalance between valleys with opposite Berry flux, yielding a nonzero net flux [17]. These studies have established that optically pumped gapped monolayer graphene supports chiral edge plasmons; however, the potential benefits of multilayer systems beyond monolayer Dirac-like systems remain largely unexplored.

Our central question is whether bilayer graphene can deliver quantitatively stronger and practically tunable nonreciprocal edge plasmons. Unlike monolayer graphene, bilayer graphene has a gate-tunable energy gap that arises from a perpendicular displacement field breaking layer inversion symmetry [22,23,24,25,26,27]. This tunability provides additional control to optimize the system’s optical response. Moreover, when gapped, bilayer graphene exhibits a Mexican-hat dispersion, in which the density of states near the energy gap diverges as the inverse square root of energy—unlike the moderately linear increase in monolayer graphene. Under circular pumping, this feature implies that bilayer graphene can support a larger valley population imbalance.

In this study, we demonstrate that these unique features of bilayer graphene lead to significantly larger and more resolvable edge-mode splitting. We modeled the nonequilibrium carrier distribution under continuous circular pumping using a density-matrix approach and computed the corresponding nonequilibrium optical conductivity. Applying the Wiener–Hopf technique, we provide an exact analysis of edge plasmons, presenting the nonreciprocal dispersion of edge plasmons, the electric potential profile, and the confinement length. While our monolayer results reproduce the previously reported chiral-plasmon phenomenology, bilayer graphene exhibits much stronger nonreciprocal splitting: for comparable gap sizes, the required pump intensities are nearly an order of magnitude lower to resolve the frequency difference between counterpropagating modes. Furthermore, the gate-controllable energy gap of bilayer graphene enhances or suppresses nonreciprocity depending on pump strength. In addition, in previous theoretical work, the demonstration of nonreciprocal edge plasmons is shown under a large energy gap (∼0.5eV) and a strong pumping field with the electric field amplitude reaching $10^{3}\;\mathrm{kV}/\mathrm{Vm}$ [17]. Our work, by contrast, not only extends the scope to bilayer graphene but also considers smaller energy gaps and much weaker pump intensities (e.g., 10kV/cm) that are more readily achievable for on-chip photonic devices. We show that, in this parameter range relevant to on-chip devices, the nonreciprocal splitting is still sizable. Our findings identify optically pumped bilayer graphene as a versatile platform for tunable, magnet-free, nonreciprocal plasmonics and motivate experimental exploration of chiral edge plasmons in gate-tunable bilayer graphene devices.

## 2. Theoretical Framework

### 2.1. Edge Plasmons

**Figure 1:**
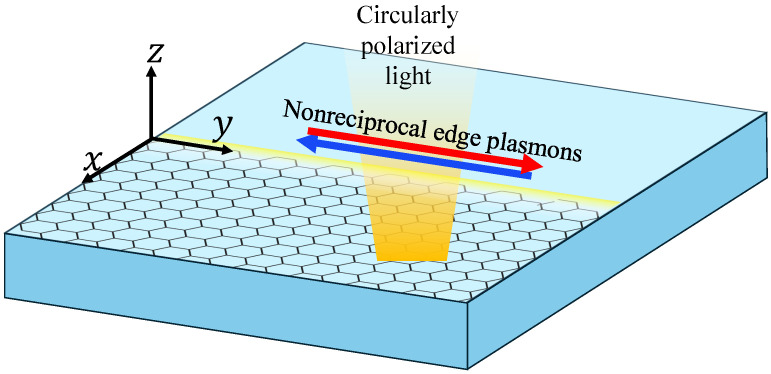
Schematic illustrations of gapped monolayer and bilayer graphene systems illuminated by circularly polarized light with red and blue arrows indicating propagation directions of nonreciprocal edge plasmon modes.

We consider a semi-infinite two-dimensional graphene sheet strictly confined to the z=0 plane and occupying the x>0 region, with edge plasmons propagating along the y-axis (see Figure 1). The two-dimensional conductivity tensor of the system, which exhibits an abrupt jump at x=0 at the boundary of the graphene sheet, is expressed as follows:

(1) $$\sigma_{ij}=\sigma_{ij}\Theta \left(x\right)\delta \left(z\right),$$

where Θ(x) is the Heaviside step function and δ(z) is the Dirac delta function. Assuming a plane wave dependence, the edge plasmon has an electric potential of the following form:

(2) $$\varphi \left(x,y,z,t\right)=\varphi \left(x,z\right)e^{i(qy-\omega t)}.$$

The associated oscillating charge density, strictly confined to the z=0 plane, is expressed as follows: $\varrho \left(x,y,z,t\right)=\varrho \left(x\right)\delta \left(z\right)e^{i(qy-\omega t)}$, where ϱ(x)=0 for x<0. The electrical potential and charge density are related through the continuity equation $\partial_{t}\varrho +\nabla \cdot J=0$, where J is the current density. Using Ohm’s law $J_{i}=\sigma_{ij}E_{j}$ and E=−∇φ, where E is the electric field, the continuity equation becomes $\partial_{t}\varrho -\sum \partial_{i}\left(\sigma_{ij}\partial_{j}\varphi \right)=0$. By substituting Equations (1) and (2) into the continuity equation, we obtain $i\omega \varrho \left(x\right)=-\delta \left(x\right)\left(\sigma_{xx}+i\sigma_{xy}q\right)\varphi \left(x\right)+\left(q^{2}\sigma_{yy}-\sigma_{xx}\partial_{x}^{2}\right)\varphi \left(x\right)-iq\partial_{x}\left(\sigma_{xy}+\sigma_{yx}\right)\varphi \left(x\right)$, where φ(x)≡φ(x,z=0), Given that $\sigma_{xx}=\sigma_{yy}$ and $\sigma_{xy}=-\sigma_{yx}$, owing to the system’s isotropy and broken time-reversal symmetry under circularly polarized light, the equation for ϱ(x) is simplified to the following:

(3) $$i\omega \varrho \left(x\right)=-\delta \left(x\right)\left(\sigma_{xx}+i\sigma_{xy}q\right)\varphi \left(x\right)+\sigma_{xx}\left(q^{2}-\partial_{x}^{2}\right)\varphi \left(x\right).$$

The term δ(x) arises from the discontinuity of the system at the boundary x=0, where the conductivity tensor changes abruptly. In the non-retarded regime q≫ω/c, the electric potential is determined by the Poisson equation $\nabla^{2}\varphi =-\frac{\varrho }{\epsilon_{b}}$, where $\epsilon_{b}$ is the effective background permittivity. At z=0, this reduces to the following integral form:

(4) $$\varphi \left(x\right)=\frac{1}{\epsilon_{b}}\int_{0}^{\infty }dx\prime L\left(x-x\prime \right)\varrho \left(x\prime \right),$$

where

(5) $$L\left(x\right)=\int_{-\infty }^{\infty }\frac{e^{ikx}}{2\sqrt{k^{2}+q^{2}}}=\frac{1}{2\pi }K_{0}\left(q\left|x\right|\right)$$

is the kernel of the integral equation and $K_{0}$ is the modified Bessel function of the second kind [12,14].

The exact electric potential and dispersion of edge plasmons can be obtained by solving Equations (3) and (4) using the Wiener–Hopf method, which is well-suited for problems with boundary conditions in a semi-infinite domain, as is the case here. The basic idea is that the real-space integral and differential equations are Fourier transformed, and the kernel of the transformed equation is factorized into two new functions, one of which is analytic in the upper half of the complex plane and the other in the lower half. This factorization separates the equation into two parts, each of which is valid on a different half-plane. This is solved using the arguments of analytic continuation. For technical details of the derivation, interested readers are referred to the existing literature [28,29,30,31,32,33,34]. In this paper, we present only the final expressions for the electrostatic potential and dispersion relation obtained using the Wiener–Hopf technique. The edge plasmon dispersion $\omega_{p}\left(q\right)$ is determined from the following nonlinear equation [34]:

(6) $$\frac{1+\lambda }{1-\lambda }\exp\frac{2i}{\pi }f\left(\lambda \right)=\frac{\pm \chi +\eta }{\pm \chi -\eta }$$

where $\eta =q\sigma_{xx}/i\epsilon_{b}\omega$ and $\chi =q\sigma_{xy}/\epsilon_{b}\omega$ are dimensionless longitudinal and Hall conductivities, respectively. Here $\lambda =-2i/\eta +\sqrt{1-{\left(2/\eta \right)}^{2}}$ and $f\left(z\right)=-\pi^{2}/6+\log\left(z\right)\log\left(1-z\right)+{\mathrm{Li}}_{2}\left(-z\right)+{\mathrm{Li}}_{2}\left(1-z\right)$, where ${\mathrm{Li}}_{2}\left(z\right)$ is the polylogarithm function of order 2. The electric potential function of an edge plasmon in the transverse plane $\varphi_{\pm }\left(x,z\right)$, where $\varphi_{+}$ corresponds to the region x>0 and $\varphi_{-}$ to x<0, is given by the following expressions [35]:

(7) $$\frac{\varphi_{+}\left(x,z\right)}{\varphi_{0}}=F_{+}\left(i\right)\frac{-i}{\varepsilon \prime \left(\xi_{2}\right)F_{-}\left(\xi_{2}\right)\left(\xi_{2}^{2}+1\right)}+F_{+}\left(i\right)\frac{\eta }{2\pi }\int_{1}^{\infty }duI_{+}\left(u\right)e^{-uqx}$$

(8) $$\begin{array}{c} \frac{\varphi_{-}\left(x,z\right)}{\varphi_{0}}=F_{+}\left(i\right)\frac{\eta }{2\pi }\int_{1}^{\infty }duI_{-}\left(u\right)e^{uqx}, \end{array}$$

where

(9) $$I_{+}\left(u\right)=\frac{\sqrt{u^{2}-1}\cos(\sqrt{u^{2}-1}q|z|)+\frac{\eta }{2}\left(u^{2}-1\right)\sin(\sqrt{u^{2}-1}q|z|)}{F_{-}\left(-iu\right)\left[u^{2}-1+\frac{1}{4}\eta^{2}{\left(u^{2}-1\right)}^{2}\right]},$$

(10) $$I_{-}\left(u\right)=\frac{\cos(\sqrt{u^{2}-1}q|z|)}{F_{+}\left(iu\right)\sqrt{u^{2}-1}}.$$

Here, $\varphi_{0}\equiv \varphi \left(0,0\right)$, $\xi_{1}$ and $\xi_{2}$ are defined as the complex roots of the two-dimensional dielectric function $\varepsilon \left(\xi \right)=1-\frac{\eta }{2}\sqrt{1+\xi^{2}}$ with $\xi_{1}$($\xi_{2}$) having a positive (negative) imaginary part, and ε′(ξ)=dε/dξ. Here, we denote by $F_{\pm }\left(\xi \right)$ the upper/lower half-plane factors of the kernel with $\varepsilon \left(\xi \right)=F_{+}\left(\xi \right)/F_{-}\left(\xi \right)$. $F_{\pm }\left(\xi \right)$ are explicitly written as $F_{+}\left(\xi \right)=\sqrt{\frac{\eta }{2}}\frac{\xi -\xi_{2}}{\sqrt{1-i\xi }}G_{+}\left(\xi \right)$ and $F_{-}\left(\xi \right)=-\sqrt{\frac{2}{\eta }}\frac{\sqrt{1+i\xi }}{\xi -\xi_{1}}G_{-}\left(\xi \right)$ with the following:

(11) $$G_{\pm }\left(\xi \right)=\exp\frac{1}{\pi }\int_{1}^{\infty }\frac{du}{i\xi \mp u}\arctan\frac{2\sqrt{u^{2}-1}}{\eta (u^{2}+1)}.$$

Throughout we use the principal branches for the square roots.

Alternatively, we can use the Fetter approximation to obtain the edge plasmon dispersion, where the integral kernel L(x) [Equation (5)] is replaced by $L\left(x\right)=\frac{K_{0}\left(q\left|x\right|\right)}{2\pi }$ with the same area and second momentum [12]. This yields the simple equation for the dispersion relation of the edge plasmon, given by $\eta^{2}-\chi^{2}-3\eta \pm 2\sqrt{2}\chi =0$ where the +(−) sign in the last term corresponds to the $\omega_{+}$($\omega_{-}$) plasmon edge mode propagating in the left (right) direction [see Figure 1]. In the low-frequency and clean limit, the longitudinal optical conductivity exhibits typical Drude behavior $\sigma_{xx}\left(\omega \right)=\frac{Di}{\omega }$, where *D* is the Drude weight, and the Hall conductivity $\sigma_{xy}$ is approximated as a constant independent of ω. Using these, it is straightforward to obtain the edge plasmon dispersion in the long-wavelength limit (q→0), which is expressed as follows:

(12) $$\omega \left(q\right)=\omega_{\mathrm{bulk}}\left(q\right)\mp \frac{\sqrt{2}\sigma_{xy}}{9\epsilon_{b}}q+\ldots ,$$

where $\omega_{\mathrm{bulk}}\left(q\right)=\sqrt{\frac{D}{3\pi \epsilon_{b}}q}$ is the bulk plasmon dispersion.

Notably, Equations (6) and (12) highlight the central role of optical conductivity in determining the nonreciprocity of the edge plasmon. In particular, the Hall component of the conductivity $\sigma_{xy}$ breaks the symmetry between modes traveling in opposite directions, which is directly responsible for the nonreciprocal propagation. Accordingly, in the next section, we present a theoretical model for the conductivity of graphene under circularly polarized light.

### 2.2. Graphene Model

Our analysis is based on a low-energy effective Hamiltonian for gapped monolayer and bilayer graphene, which captures the essential features of low-energy physics near the K and K′ valleys. The Hamiltonians for gapped monolayer and Bernal-stacked bilayer graphene with an energy gap *U* are given by $H_{\tau }^{\mathrm{MLG}}\left(k\right)=\hbar v_{F}\left(\sigma \cdot k_{\tau }\right)+\frac{U}{2}\;\sigma_{z}$ and $H_{\tau }^{\mathrm{BLG}}\left(k\right)=\hbar v_{F}\left[\mu_{0}⊗\left(\sigma \cdot k_{\tau }\right)\right]+\frac{U}{2}\left(\mu_{z}⊗\sigma_{0}\right)+\frac{t}{2}\left(\mu_{x}⊗\sigma_{x}-\mu_{y}⊗\sigma_{y}\right)$, respectively, where $k_{\tau }=\left(\tau k_{x},k_{y}\right)$ is the momentum vector for valley index τ=±1, $v_{F}$ is the Fermi velocity of graphene, *U* is the interlayer potential bias, and *t* is the interlayer hopping energy [36,37]. The matrices $\sigma =(\sigma_{x},\sigma_{y},\sigma_{z})$ and $\mu =(\mu_{x},\mu_{y},\mu_{z})$ are Pauli matrices for sublattice and layer pseudospin, respectively, while $\sigma_{0}$ and $\mu_{0}$ denote 2×2 identity matrices.

### 2.3. Steady-State Charge Distribution and Optical Conductivity

Under continuous optical pumping, the carrier distribution reaches a time-independent steady-state nonequilibrium. The steady-state distribution function is obtained by solving the von Neumann equation, which describes the evolution of the density matrix under the influence of an electromagnetic field: $i\hbar \frac{\partial \hat{\rho }}{\partial t}=\left[{\hat{H}}_{0}+\hat{V},\hat{\rho }\right]$ where $H_{0}$ is the bare Hamiltonian without interaction with the electromagnetic field, $\hat{\rho }$ is the density matrix, and V=eE·r is the interaction term that considers coupling with the electromagnetic field with $E=E_{0}e^{i\omega t}+c.c$ being the electric field of the circularly polarized monochromatic pumping light. Using the rotating-wave and relaxation-time approximations, the diagonal components of the density matrix are obtained by solving the following system of linear equations [17]:

(13a)ρvvK=βK(ρvveq,K+γρvvK′+αKρccK),(13b)ρvvK′=βK′(ρvveq,K′+γρvvK+αK′ρccK′),(13c)ρccK=βK(ρcceq,K+γρccK′+αKρvvK),(13d)ρccK′=βK′(ρcceq,K′+γρccK+αK′ρvvK′),

where $\alpha_{\tau }=\frac{2e^{2}\tau_{0}\tau_{d}{\left|E_{0}\cdot R_{\mathrm{cv}}^{\tau }\right|}^{2}}{\hbar^{2}\left[\tau_{d}^{2}{\left(\omega -\omega_{cv}\right)}^{2}+1\right]}$, $\beta_{\tau }=1/\left(1+\alpha_{\tau }+\gamma \right)$, $\hbar \omega_{\mathrm{cv}}=E_{c}-E_{v}$, and $\gamma =\tau_{0}/\tau_{1}$ with $\tau_{0}$ and $\tau_{1}$ denoting the electron-hole and valley relaxation times, respectively. $\tau_{d}$ is the dephasing time required for decoherence between the conduction and valence bands and $R_{\mathrm{cv}}^{\tau }=i\langle u_{ck}^{\tau }\;|\;\nabla_{k}u_{vk}^{\tau }\rangle ,$ is the valley-dependent dipole matrix element that determines the optical selection rule, where $|u_{s\prime ,k}^{\tau }\rangle$ is the electronic wavefunction and $E_{c(v)}$ is the lowest conduction (highest valence) energy band. Unless otherwise specified, all numerical results were obtained with realistic parameter values: $\tau_{0}=\tau_{1}=1\;\mathrm{ps}$ and $\tau_{d}=50\;\mathrm{fs}$. Without loss of generality, we assume right circularly polarized light (i.e., $E_{0}=E_{0}\left(\hat{x}+i\hat{y}\right)/\sqrt{2}$) where $E_{0}$ denotes the pumping field amplitude.

**Figure 2:**
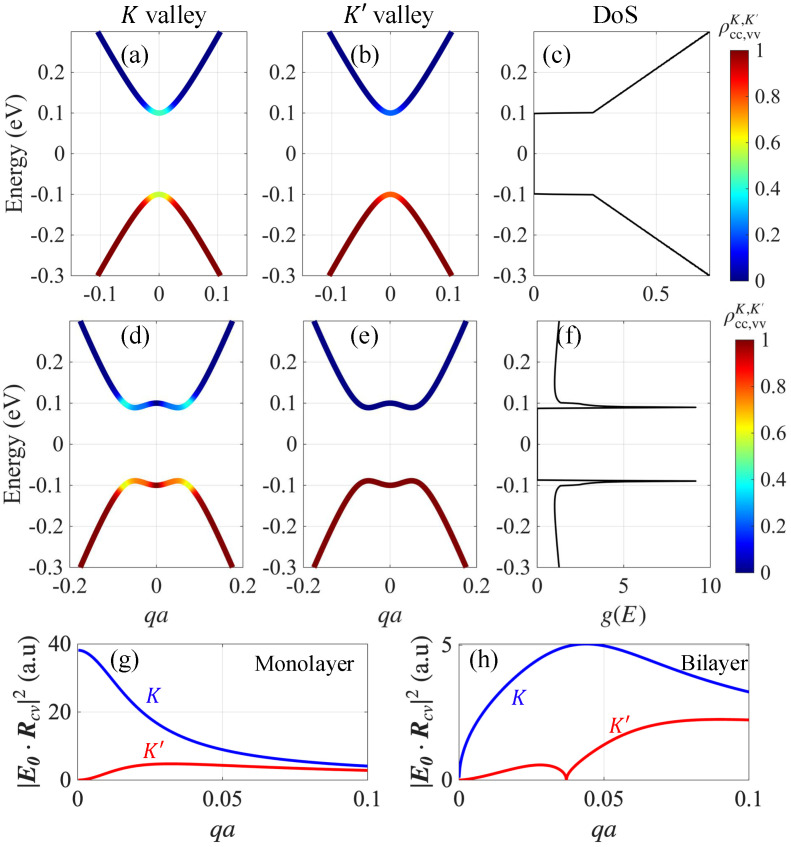
Energy-band dispersions for (**a**,**b**) gapped monolayer and (**d**,**e**) bilayer graphene at the K and K′ valleys. The color scale shows the steady-state carrier population under circular pumping, highlighting the valley-dependent selection rule. The corresponding density of states for monolayer and bilayer graphene is shown in panels (**c**) and (**f**), respectively. Numerically calculated $\left|E_{0}\cdot R_{\mathrm{cv}}^{\tau }\right|$ for (**g**) gapped monolayer and (**h**) bilayer graphene for both K (blue) and K′ (red) valleys, which shows the optical selection rule. The calculations are performed with an energy gap of U=0.2eV and a pump field strength of $E_{0}=10\;\mathrm{kV}/\mathrm{cm}$. For clarity of presentation, we set the temperature to zero here to eliminate thermal occupations. Here we use the normalized wavevector qa where *a* is the graphene lattice constant.

**Figure 3:**
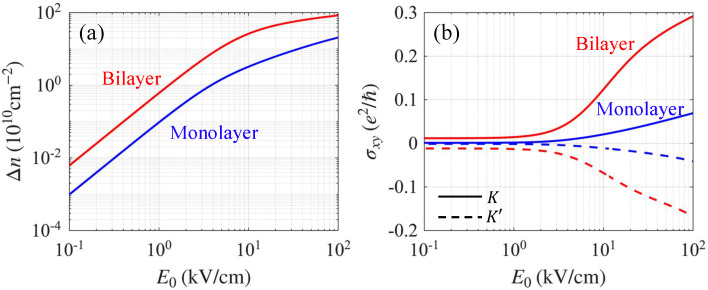
(**a**) Photoinduced valley population imbalance ($\Delta n=n^{K}-n^{K\prime }$) as a function of $E_{0}$ for gapped monolayer (blue) and bilayer (red) graphene. (**b**) Static photo induced Hall conductivity per valley (K: solid; K′: dashed), including both electron and hole contributions.

Figure 2 shows the numerically calculated steady-charge distribution functions at K and K′ for both monolayer and bilayer graphene with a finite energy gap of U=0.2eV. The pump frequency was chosen to match the energy gap to maximize the optical pumping effects, and the temperature was set to T=0 to clearly reveal valley-polarized optical excitation. Unless otherwise stated, the remainder of this study uses a room temperature of T=300K. The selection rule is captured in Figure 2g,h which plot the magnitude of the field-projected dipole matrix element, $\left|E_{0}\cdot R_{\mathrm{cv}}^{\tau }\right|$. For monolayer graphene, the selection rule forbids interband optical pumping at the K′ Dirac point, whereas the selection rule in bilayer graphene forbids optical pumping at both Dirac points (i.e., K and K′ valleys), i.e., the rule is completely valley independent [38,39]. The valley-dependent selection rule reemerges at finite momentum $k=k_{0}$ around each valley, where $\varepsilon_{c}\left(k_{0}\right)-\varepsilon_{v}\left(k_{0}\right)=U$. Importantly, Bernal-stacked bilayer graphene exhibits a Mexican-hat energy dispersion, resulting in a diverging density of states near the energy gap, in contrast to gapped monolayer graphene, where the density of states increases linearly without peaks near the gap [see Figure 2c,f]. This enhanced density of states leads to more efficient optical absorption in the bilayer graphene. Consequently, more charges are pumped in the bilayer graphene for the same pumping field strength, as shown in Figure 3a, where we plot the valley imbalance $\Delta n=n^{K}-n^{K\prime }$, with $n^{K}$ and $n^{K’}$ denoting the photoexcited carrier densities at K and K′ valleys, respectively.

We take an approximate approach to calculate the optical conductivity of graphene driven by continuous circularly polarized light using the standard Kubo formula with the equilibrium Fermi–Dirac distribution replaced by the nonequilibrium distribution function evaluated using Equation (13) [40,41,42]:

$$\sigma_{ij}\left(\omega \right)=-g\frac{ie^{2}}{\hbar }\sum_{s,s\prime ,\tau }\int \frac{d^{2}k}{{\left(2\pi \right)}^{2}}\frac{\rho_{s,k}^{\tau }-\rho_{s\prime ,k}^{\tau }}{E_{s,k}-E_{s\prime ,k}}\frac{M_{i}^{\tau ,ss\prime }\left(k\right)M_{j}^{\tau ,s\prime s}\left(k\right)}{\hbar \omega +E_{s,k}^{\tau }-E_{s\prime ,k}^{\tau }+i\gamma_{0}}$$

where *g* is the spin degeneracy, $E_{s\prime ,k}^{\tau }$ is the band energy, s, s′ are band indices, i,j=x,y, and $M_{i}^{\tau ,ss\prime }\left(k\right)=\langle u_{s,k}^{\tau }|\;{\hat{v}}_{i}\;|u_{s\prime ,k}^{\tau }\rangle$. where ${\hat{v}}_{i}=\left(1/\hbar \right)\;\partial H^{\tau }/\partial k_{i}$ denotes the velocity operator. The parameter $\gamma_{0}$ is a phenomenological broadening parameter that incorporates multi-body and disorder effects. This approximation neglects coherent dressing of the electronic bands, which is justified in the weak-driving regime quantified by the dimensionless quantity $\left(A=eE_{0}a/\hbar \omega_{\mathrm{pump}}\ll 1\right)$ [43,44,45,46,47,48,49,50,51], where $\omega_{\mathrm{pump}}$ is the pump frequency. Typical experimental parameters relevant to our work (e.g., $E_{0}∼10\;\mathrm{kV}/\mathrm{cm}$, $\hbar \omega_{\mathrm{pump}}∼0.1\;\mathrm{eV}$, $A∼10^{-3}\ll 1$) ensure that we remain within the weak-driving limit. In all numerical calculations, we use a realistic broadening parameter $\gamma_{0}=10\;\mathrm{meV}$ [52,53,54], which is appropriate for moderately clean graphene. The momentum integral is carried out over $k\in [0,k_{c}]$ and θ∈[0,2π] using an adaptive quadrature method with the cutoff momentum $k_{c}$ chosen so that the conductivity barely changes upon doubling $k_{c}$. Because the integrand contains weak and integrable singularities, we enforce a minimum of $10^{6}$ subintervals in the adaptive quadrature to ensure robust convergence and accuracy.

As Hall conductivity breaks reciprocity between counterpropagating plasmon edge modes, it is instructive to first examine the photoinduced Hall conductivity before presenting our numerical results for edge plasmons. Although our full numerical calculations of the edge plasmons used the dynamic photoinduced Hall conductivity, we present the static photoinduced Hall conductivity contribution at K and K′ valleys in Figure 3. The ω dependence of the Hall conductivity is weak unless the plasmon frequency is comparable to the energy gap, so the static Hall conductivity captures the relevant trend. Note that the Hall conductivity has opposite signs owing to the opposite chirality of the K and K′ valleys. For a weak electric field strength, only a small fraction of the charges is pumped, and the Hall conductivity for both monolayer and bilayer is negligibly small. However, above $E_{0}∼3\;\mathrm{kV}/\mathrm{cm}$, $\sigma_{xy}$ in bilayer graphene grows very rapidly, reaching $10^{-1}e^{2}/\hbar$, whereas in monolayer graphene, $\sigma_{xy}$ reaches the same magnitude only at much higher field strengths $E_{0}$. Thus, bilayer graphene exhibits significantly stronger nonreciprocity than monolayer graphene.

After obtaining the optical conductivity, the next section presents the numerical results for plasmon dispersion and localization length, obtained using Equations (6)–(8) as presented in the previous section.

## 3. Results and Discussion

**Figure 4:**
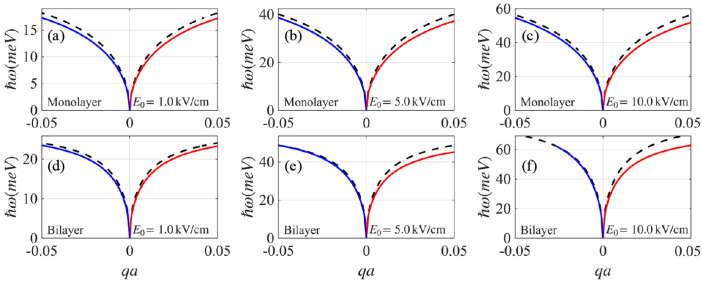
Edge plasmon dispersions for (**a**–**c**) monolayer and (**d**–**f**) bilayer graphene at $E_{0}=1$, 5, and 10kV/cm. The horizontal axis represents the in-plane wave vector projected along the edge, expressed in dimensionless form qa, while the vertical axis gives the corresponding plasmon energy in units of meV. The red (blue) line represents the right (left-)-propagating edge plasmon mode, and the black dashed line represents the two-dimensional bulk plasmon mode. Here, the energy gap U=0.2eV was used for the calculations.

Figure 4 compares the edge plasmon dispersion (solid lines) with the bulk graphene plasmon (black dashed line) for monolayer [panels (a–c)] and bilayer [panels (d–f)] graphene as the pump amplitude increases from $E_{0}=1$, 5, to 10kV/cm. For the weakest pump amplitude, $E_{0}=1.0\;\mathrm{kV}/\mathrm{cm}$ [panels (a) and (d)], both edge plasmon modes are displaced relative to the bulk plasmon mode by nearly the same amount, indicating weak nonreciprocity. As $E_{0}$ increases, the nonreciprocity becomes stronger: the left (right-)-propagating mode approaches (moves away from) the bulk mode. This trend follows the increase in the photoinduced Hall conductivity, as shown in Figure 3. At $E_{0}=10\;\mathrm{kV}/\mathrm{cm}$, the left-propagating branch edge mode merges with the bulk mode at a critical momentum $q_{c}$; for $q>q_{c}$, only the right-propagating mode remains, rendering the edge plasmon effectively unidirectional. We emphasize that the edge plasmon nonreciprocity is much more pronounced in bilayer graphene, consistent with its higher photoinduced Hall conductivity discussed in the previous section. In particular, achieving a comparable level of nonreciprocity in bilayer graphene requires approximately an order of magnitude smaller $E_{0}$ than in monolayer graphene.

**Figure 5:**
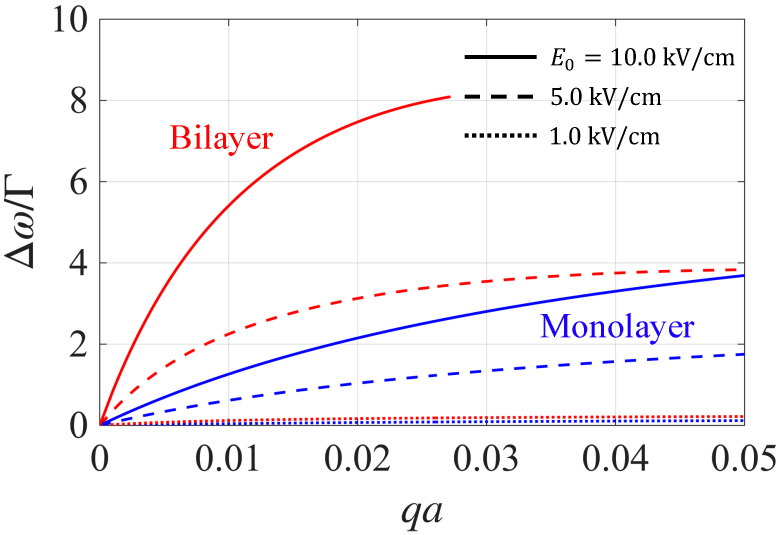
Plot of Δω/Γ as a function of qa at different values of $E_{0}=1.0$, 5.0 and 10.0kV/cm, ranging from very weak to strong pumping.

The stronger nonreciprocity in bilayer graphene is more pronounced in Figure 5, where we present the dimensionless ratio Δω/Γ for the three pump amplitudes used in Figure 4. Here $\Delta \omega =\omega_{+}-\omega_{-}$ is the plasmon frequency splitting, $\omega_{+(-)}$ denotes the right/left–propagating branches, and Γ=−2Im(ω) is the plasmon decay rate. The dimensionless ratio Δω/Γ quantifies the spectral resolvability of the two counterpropagating edge modes with Δω≫Γ, implying that their plasmon frequencies are easily resolved. For the weak pump, $E_{0}=1.0\;\mathrm{kV}/\mathrm{cm}$, Δω≪Γ in both mono- and bilayer graphene. Thus, the edge plasmon nonreciprocity is effectively unobservable. Increasing $E_{0}$ enhances Δω/Γ. In bilayer graphene, Δω/Γ is well above unity over a broad momentum range of $E_{0}=5\;\mathrm{kV}/\mathrm{cm}$. By contrast, in monolayer graphene, a larger pump amplitude $E_{0}=10\;\mathrm{kV}/\mathrm{cm}$ is required to reach Δω/Γ≳1. At the same pump amplitude ($E_{0}=10\;\mathrm{kV}/\mathrm{cm}$), Δω/Γ reaches ∼8 in bilayer graphene, significantly larger than that for monolayer graphene. The bilayer curves terminate at the critical momentum $q_{c}$, where the left–propagating edge mode merges with the bulk plasmon and ceases to exist, consistent with the onset of unidirectionality.

**Figure 6:**
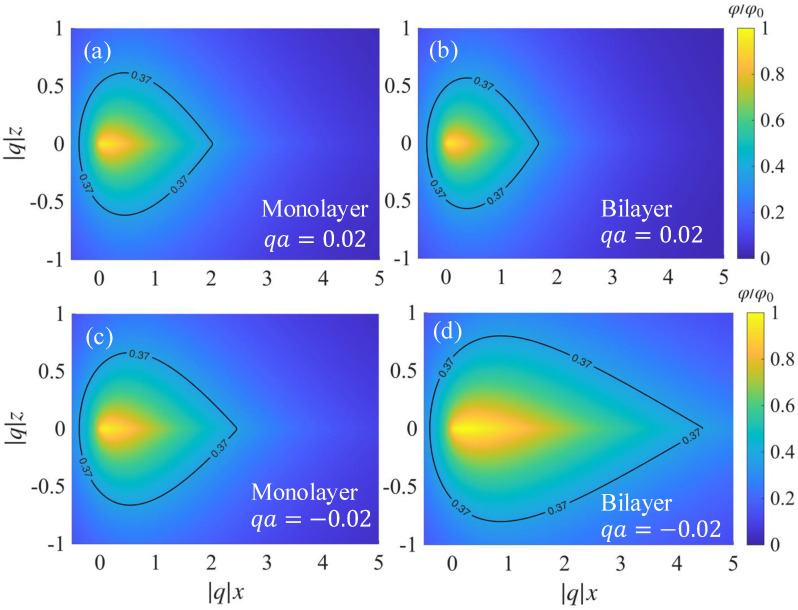
Spatial profile of the normalized electrostatic potential for edge plasmons at $\left|q\right|a=0.02$ and $E_{0}=10\;\mathrm{kV}/\mathrm{cm}$ for (**a**,**b**) right- and (**c**,**d**) left-propagating modes. The contour plot is drawn for $\varphi /\varphi_{0}=1/e\approx 0.37$.

In Figure 6, we plot the spatial profile of the normalized electrostatic potential $\varphi /\varphi_{0}$ of the edge plasmons propagating in monolayer (a,c) and bilayer (b,d) graphene in the transverse plane ($\left|q\right|x,\left|q\right|z$) at fixed $\left|q\right|a=0.02$. The black curve corresponds to the $\varphi /\varphi_{0}=1/e$ contour and serves as a measure of transverse confinement. A comparison of panels (a) and (c) with panels (b) and (d) shows that the potential profile depends on the propagation direction, indicating that nonreciprocity manifests not only in the dispersion but also in the transverse confinement length. For qa=0.02, both monolayer and bilayer edge modes are tightly localized with nearly identical confinement lengths, and the bilayer is only slightly more localized. This observation is consistent with the monolayer and bilayer edge-plasmon dispersions being well-separated from the bulk plasmon dispersion for qa>0. In contrast, for qa=−0.02, which is close to the critical momentum $q_{c}$ where the bilayer edge and bulk plasmon modes merge, the bilayer mode substantially broadens into the interior, exhibiting a much longer confinement length. The monolayer mode is well-localized, similar to the qa>0 case, because the plasmon dispersion is well-displaced from the bulk mode. We emphasize that the overlap between the counterpropagating edge fields is much smaller in the bilayer, implying that elastic backscattering of edge plasmons by imperfections is more strongly suppressed in the bilayer graphene [16,55,56,57].

**Figure 7:**
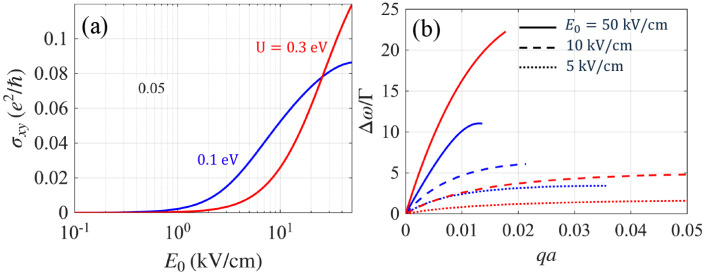
(**a**) Plots of the photoinduced Hall conductivity $\sigma_{xy}$ as a function of $E_{0}$ in units of $e^{2}/\hbar$ for two different values of U=0.1 and 0.3eV. (**b**) The resolvability metric Δω/Γ for $E_{0}=5$, 10, and 50kV/cm.

In dual-gated bilayer graphene, a perpendicular displacement field breaks the inversion symmetry and induces an interlayer potential asymmetry *U*, allowing continuous gate tuning of the energy band gap [22,23,24,25,26,27]. Accordingly, we examine how the tunable interlayer bias *U* affects edge-plasmon nonreciprocity. In Figure 7a, we plot the photoinduced Hall conductivity as a function of pump amplitude $E_{0}$ for U=0.1 and 0.3eV. As established above, edge-mode nonreciprocity increases with photoinduced Hall conductivity $\sigma_{xy}$; thus, Figure 7a provides a practical guide to how nonreciprocity evolves with *U*. For a weak field, $E_{0}≲1\;\mathrm{kV}/\mathrm{cm}$, $\sigma_{xy}$ is larger for the smaller gap. However, with increasing $E_{0}$, $\sigma_{xy}$ increases more rapidly for the larger *U*, and the ordering reverses at $E_{0}>20\;\mathrm{kV}/\mathrm{cm}$. This behavior carries over to the edge-mode splitting shown in Figure 7b, where Δω/Γ is plotted as a function of qa for fixed values of $E_{0}=5$, 10, and 50kV/cm. Note that, for $E_{0}=5$ and 10kV/cm, Δω/Γ follows the same order as $\sigma_{xy}$. For a strong pump amplitude of $E_{0}=50\;\mathrm{kV}/\mathrm{cm}$, the ordering reverses with Δω/Γ, which is almost twice as large as that for U=0.1eV. In summary, our analysis shows that large nonreciprocity can be achieved with a large energy gap if a large $E_{0}$ is available. However, for weak $E_{0}$, a small energy gap is more favorable for producing nonreciprocal edge plasmons. Our results show that, for sufficiently large pump amplitudes, increasing the gate-induced gap *U* enhances the nonreciprocal splitting Δω/Γ, whereas in the low-field regime, a smaller gap yields a larger Δω/Γ.

Our analysis so far assumes that the pump photon energy is resonant with the band gap *U*, which we vary between 0.1 and 0.3eV. This mid-infrared band is well covered by established sources such as quantum cascade lasers (QCLs) [58,59,60,61,62], which are already mature for room-temperature continuous-wave operation across this range. Moreover, on-chip integration of QCLs has been shown via transfer-printing and heterogeneous bonding onto Si/Ge-on-Si platforms, and QCLs have been used to excite graphene on a chip-scale mid-IR device [58,59]. It should be noted, however, that operation beyond 0.2eV can be problematic due to heating effects such as rapid optical-phonon emission [63,64], resulting in shorter valley relaxation times. While operation up to 0.3eV still remains feasible with pulsed or low-duty excitation and proper thermal management, a practical upper is approximately <0.2eV so that coupling to optical phonons is suppressed.

We end this section with a discussion on the role of stacking order on the edge plasmon nonreciprocity. For AA stacked bilayer graphene, the electronic band structure is gapless, with two Dirac cones shifted in energy by the interlayer hopping [65]. A perpendicular electric field does not open an energy gap, only pushing the two Dirac cones further apart in energy. In such metallic cases, where the Berry curvature (and thus the Hall conductivity) is negligible [66], the nonreciprocity of edge plasmons is expected to be extremely small, in contrast to the AB-stacked bilayer graphene studied in our work. For BA stacking, reversing the stacking from AB to BA at a fixed energy gap should flip the sign of the Hall conductivity and therefore reverse the direction of nonreciprocity. Furthermore, this result for BA stacking indicates that an AB-BA stacking domain wall under a perpendicular electric field flips the sign of the Hall conductivity across the interface. In our electrodynamic formulation, this acts as a 1D boundary with a discontinuous conductivity tensor [c.f., Equation (1)], yielding a guided domain-wall plasmon. The details of this plasmon mode can be obtained at the same approximation level as our edge-mode analysis and are left for future work.

## 4. Conclusions

We investigated edge plasmons in gapped monolayer and bilayer graphene under continuous circularly polarized light. Optical pumping induces a population imbalance between valleys, and the resulting photoinduced Hall conductivity breaks the reciprocity of counterpropagating plasmon edge modes without requiring a magnetic field. Using the exact Wiener–Hopf method, we calculated the edge plasmon dispersion, confinement length, and spatial profile of the plasmonic edge-mode electric field, demonstrating that circular pumping in gapped bilayer graphene also induces strong nonreciprocity in edge plasmons predicted for gapped Dirac materials. We found that bilayer graphene, owing to its Mexican-hat electronic band structure, supports significantly stronger nonreciprocal splitting, achieving resolvable chiral splitting (Δω/Γ≳1) at pump fields nearly an order of magnitude weaker than those required for gapped monolayer graphene. Our results further indicate that increasing the interlayer bias *U* of bilayer graphene enhances the plasmon edge-mode nonreciprocity in strong fields but reduces it in weak fields. Because *U* is gate-tunable, this additional control provides a practical means of optimizing nonreciprocity in bilayer-graphene plasmonic devices for a given pump. Overall, these findings suggest that optically pumped bilayer graphene is a promising candidate for compact, tunable, and nonreciprocal plasmonic devices operating in the mid-infrared regime.

## Data Availability

The original contributions presented in this study are included in the article. Further inquiries can be directed to the corresponding author.
